# Therapeutic advances in hepatocellular carcinoma: an update from the 2024 ASCO annual meeting

**DOI:** 10.3389/fonc.2024.1453412

**Published:** 2024-10-25

**Authors:** Hongyuan Yang, Yanju Liu, Na Zhang, Fengbao Tao, Gaozheng Yin

**Affiliations:** Department of Infectious Diseases, Weifang People’s Hospital, Weifang, Shandong, China

**Keywords:** hepatocellular carcinoma, 2024 ASCO meeting, immunotherapy, targeted therapy, combinational therapy

## Abstract

Hepatocellular carcinoma (HCC) remains a leading cause of cancer-related deaths worldwide. Recent advances in immunotherapies, targeted therapies, and combination treatments have significantly improved outcomes for many patients with HCC. This review summarizes key findings from the 2024 ASCO Annual Meeting, focusing on emerging therapies, including immune checkpoint inhibitors (ICIs), CAR-T cell therapies, oncolytic viruses, and locoregional treatments like transarterial chemoembolization (TACE) and hepatic arterial infusion chemotherapy (HAIC). ICIs, particularly when combined with other agents, have shown promising efficacy, though challenges such as immune-related adverse events and resistance mechanisms remain. CAR-T cell therapies and oncolytic viruses offer novel therapeutic avenues for advanced HCC, but their long-term efficacy in solid tumors is still under investigation. Locoregional therapies, especially in combination with systemic treatments, continue to play a critical role in managing unresectable HCC and improving conversion rates to surgical resection. Additionally, the potential of biomarkers, such as hypoxia scores and CTNNB1 mutations, is being explored to better personalize treatment and predict patient responses. These biomarkers could pave the way for more targeted and effective therapeutic strategies. Overall, the recent studies presented at the ASCO meeting highlight progress in HCC treatment, underscoring the importance of continued innovation. Future research should focus on overcoming resistance mechanisms, optimizing combination therapies, and integrating biomarker-driven approaches to improve patient outcomes and enhance personalized treatment strategies.

## Introduction

Hepatocellular carcinoma (HCC) is the most common primary liver cancer, making up 75% of cases (1). It is the sixth most common cancer and the fourth leading cause of cancer deaths globally (2). Despite advances in the early detection and treatment of HCC, the prognosis for many patients remains poor, largely due to late diagnosis and the aggressive nature of the disease (3). Current standard-of-care treatments, including surgical resection, liver transplantation, and locoregional therapies such as transarterial chemoembolization (TACE) and radiofrequency ablation (RFA), are primarily effective in patients with early-stage or localized tumors (4, 5). Current standard-of-care treatments, including surgical resection, liver transplantation, and locoregional therapies such as transarterial chemoembolization (TACE) and radiofrequency ablation (RFA), are primarily effective in patients with early-stage or localized tumors (6, 7).

A significant limitation of existing therapies is the development of resistance, which is frequently driven by mechanisms such as epithelial-mesenchymal transition (EMT) and cancer cell plasticity. These processes not only promote metastasis but also enhance tumor cells’ ability to evade therapeutic interventions, leading to therapy resistance and poor patient outcomes (8). Emerging evidence also suggests that nanoparticles, particularly those functionalized with peptides, offer new opportunities to enhance drug delivery and target tumor cells more effectively, potentially overcoming some of the limitations seen with traditional therapeutic agents (9).

**Figure 1:**
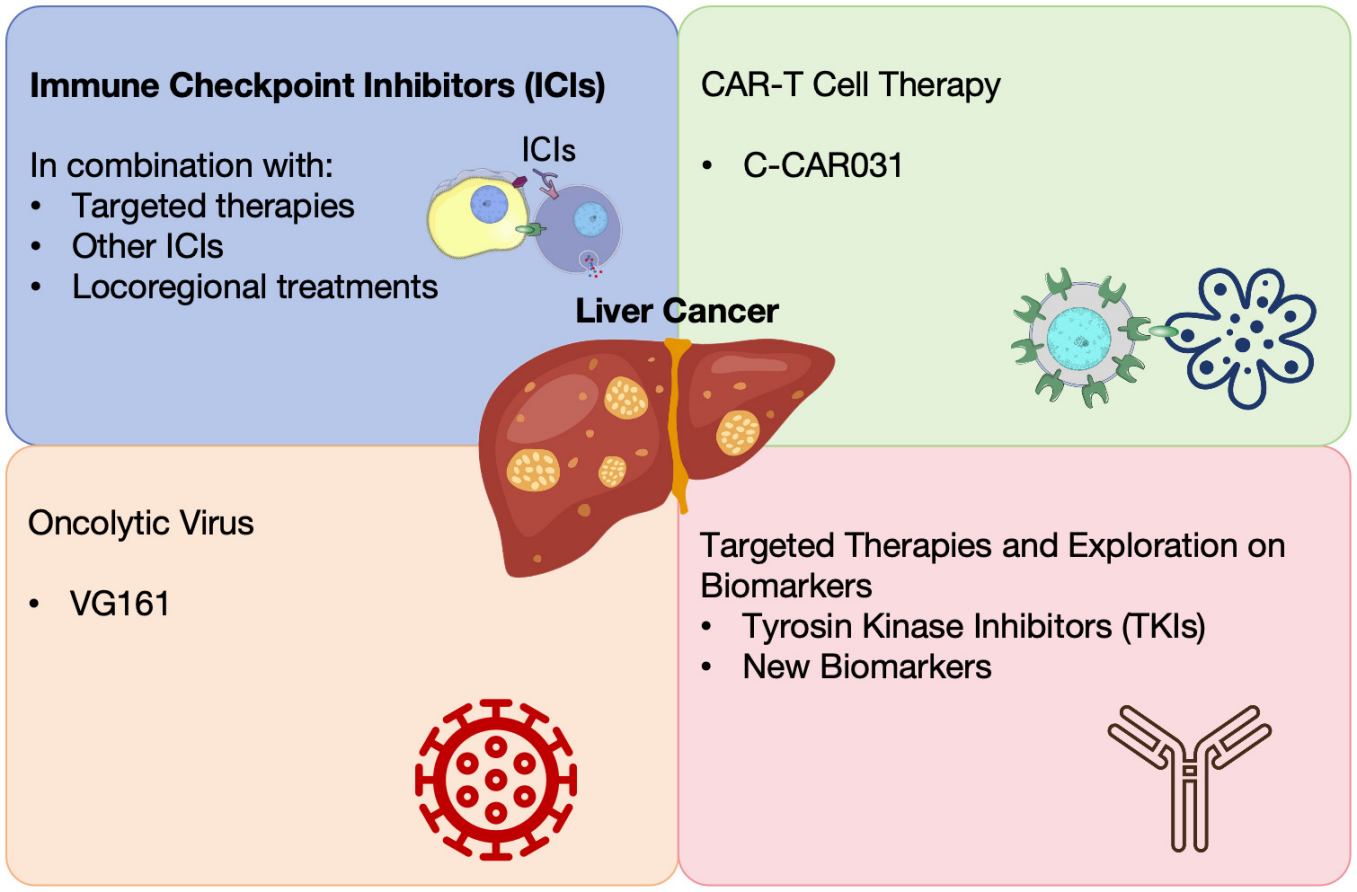
Summary of the therapeutic advances in HCC reported in 2024 ASCO annual meeting.

The landscape of HCC treatment has been revolutionized by the advent of immune checkpoint inhibitors (ICIs), targeted therapies, and novel combinations (10) (**Figure 1**). Different strategies including targeting the tumor-infiltrating neutrophils and using oncolytic viruses have been explored to enhance the efficacy of liver cancer immunotherapy (11, 12). These advancements offer new hope for improved survival and quality of life for patients with advanced HCC. These offer hope for better survival and quality of life in advanced HCC. We summarized 34 clinical studies on liver cancer from the 2024 ASCO meeting, highlighting progress in treatments like ICIs, locoregional therapies, CAR-T cell therapies, and more (**Table 1**).

**Table 1:** Clinical trials reported in 2024 ASCO Annual Meeting.

Abstract number	Name of study	NCT Number	Phase	Offical Title	Sample Size(n)	Conditions	Outcome Measures
4007		NCT04696055	Phase 2	Regorafenib Plus Pembrolizumab in Patients With Advanced or Spreading Liver Cancer Who Have Been Previously Treated With PD-1/​PD-L1 Immune Checkpoint Inhibitors	95	Hepatocellular Carcinoma	1.ORR 2.CR 3.PR
4008	CheckMate 9DW	NCT04039607	Phase 3	A Study of Nivolumab in Combination With Ipilimumab in Participants With Advanced Hepatocellular Carcinoma (CheckMate 9DW)	732	Hepatocellular Carcinoma	1.OS
4019	C-CAR031	NCT05155189	Phase 1	A Study to Evaluate Safety and Efficacy of Armored CAR-T Cell Injection C-CAR031 in Advanced Hepatocellular Carcinoma	44	Hepatocellular Carcinoma	1.TEAEs 2.AESIs
4022		NCT02519348	Phase 2	A Study of Durvalumab or Tremelimumab Monotherapy, or Durvalumab in Combination With Tremelimumab or Bevacizumab in Advanced Hepatocellular Carcinoma	433	Hepatocellular Carcinoma	1.DLTs 2.TEAEs 3.CTCAE 4.ECG
4026				RNA expression-based hypoxia score as a prognostic and predictive biomarker in hepatocellular carcinoma	432	Hepatocellular carcinoma	1.OS
4092	SCT-I10A	NCT04560894	Phase 2, Phase 3	SCT-I10A Plus SCT510 Versus Sorafenib as First-Line Therapy for Advanced Hepatocellular Carcinoma (HCC)	405	Hepatocellular Carcinoma	1.OS 2.PFS
4094	REFLECT	NCT01761266	Phase 3	A Multicenter, Open-Label, Phase 3 Trial to Compare the Efficacy and Safety of Lenvatinib (E7080) Versus Sorafenib in First-line Treatment of Participants With Unresectable Hepatocellular Carcinoma	954	Hepatocellular Carcinoma	1.OS
4096				Survival outcomes with adjuvant immunotherapy after hepatic resection in patients with intermediate/advanced (BCLC stage B/C) hepatocellular carcinoma: Insights from a propensity-matched multicenter study	627	hepatocellular carcinoma	1.RFS 2.OS
4098				Predictors of short-term death and long-term survival in advanced hepatocellular carcinoma (HCC) treated with contemporary tyrosine kinase inhibitors (TKI) or immunotherapy: A multicenter analysis from the HCC-CHORD consortium	520	hepatocellular carcinoma	1.STD 2.LTS
4099				Atezolizumab plus bevacizumab (A+B) as first-line systemic therapy for advanced hepatocellular carcinoma (HCC): A multi-institution analysis of patient outcomes based on Child Pugh (CP) and ALBI liver function	322	hepatocellular carcinoma	1.PFS 2.OS
4100	KEYNOTE-224	NCT02702414	Phase 2	Study of Pembrolizumab (MK-3475) as Monotherapy in Participants With Advanced Hepatocellular Carcinoma (MK-3475-224/​KEYNOTE-224)	156	Hepatocellular Carcinoma	1.ORR
4102				Stereotactic body radiotherapy (SBRT) combined with transcatheter arterial che moembolization (TACE) and tyrosine kinase inhibitors (TKIs) versus TACE and TKIs alone for unresectable hepatocellular carcinoma (uHCC) with portal vein tumor thrombus (PVTT): A randomized controlled trial	90	unresectable hepatocellular carcinoma	1.ORR 2.OS 3.DCR 4.TRAE
4103			Phase 2	Hepatic artery infusion chemotherapy (HAIC) combined with tislelizumab and lenvatinib for initial unresectable hepatocellular carcinoma (HCC) with portal vein tumor thrombus: A prospective, single-arm phase II trial	29	unresectable hepatocellular carcinoma	1.ORR 2.DCR 3.PFS 4.OS 5.TRAE
4105		NCT04806464	Phase 1	Clinical Study of VG161 in Subjects With Advanced Primary Liver Cancer	44	Primary Liver Cancer	1.MTD 2,DLT 3.AE 4.SAE 5.ORR
4106				Impact of CTNNB1 alterations on outcomes in patients with hepatocellular carci noma (HCC)		HCC	1.OS
4108		NCT05213221	Phase 2	Study on Therapeutic Effect of Combination of Envafolimab, Lenavatinib and TACE in Advanced HCC Patients (CISLD-12)	39	Advanced Hepatocellular Carcinoma	1.ORR
4109	KEYNOTE-240	NCT02702401	Phase 3	Study of Pembrolizumab (MK-3475) vs. Best Supportive Care in Participants With Previously Systemically Treated Advanced Hepatocellular Carcinoma (MK-3475-240/​KEYNOTE-240)	413	Hepatocellular Carcinoma	1.PFS 2.OS
4110	CARES-310	NCT03764293	Phase 3	A Study to Evaluate SHR-1210 in Combination With Apatinib as First-Line Therapy in Patients With Advanced HCC	543	Locally Advanced or Metastatic and Unresectable HCC	1.OS 2.PFS
4111				Atezolizumab + bevacizumab versus lenvatinib as first-line systemic therapy for treatment of hepatocellular carcinoma in a real-world population: Outcomes from the HCC CHORD database	453	Hepatocellular Carcinoma	1.OS 2. PFS 3. RR
4112		NCT05412589	Phase 2	mFOLFOX7 Plus Camrelizumab and Apatinib for Advanced HCC	35	Stage III Hepatocellular Cancer (CNLC Staging)	1.ORR
4113	SHATA-001	NCT02856126	Phase 3	HAIC Plus Sorafenib Versus TACE Plus Sorafenibfor Advanced HCC	214	Hepatocellular Carcinoma	1.OS
4118			Phase 2	Tislelizumab plus intensity modulated radiotherapy in resectable hepatocellular carcinoma with macrovascular invasion: A prospective, single-arm, phase II trial	30	Hepatocellular Carcinoma	1.ORR 2.DCR 3.OS 4.RFS
4120	NeoLEAP-HCC	NCT05389527	Phase 2	Pembrolizumab and Lenvatinib for Resectable Hepatocellular Carcinoma (NeoLeap-HCC)	43	Carcinoma, Hepatocellular	1.MPR
4122	EMERALD-1	NCT03778957	Phase 3	A Global Study to Evaluate Transarterial Chemoembolization (TACE) in Combination With Durvalumab and Bevacizumab Therapy in Patients With Locoregional Hepatocellular Carcinoma (EMERALD-1)	724	Hepatocellular Carcinoma	1.PFS
4123	PLATIC	NCT04814043	Phase 2	PD-1 Antibody and Lenvatinib Plus TACE-HAIC for Potential Resectable HCC: a Single-arm, Phase 2 Clinical Trial (PLATIC)	57	Hepatocellular Carcinoma	1.conversion rate to resection
4125				Utilization of tumor-informed circulating tumor DNA in detecting minimal residual disease and guiding adjuvant therapy in liver cancer	136	Hepatocellular Carcinoma	
4187	GEMINI-Hepatobiliary	NCT05775159	Phase 2	Study of Novel Immunomodulators as Monotherapy and in Combination With Anticancer Agents in Participants With Advanced Hepatobiliary Cancer	260	Hepatocellular Carcinoma,Biliary Tract Cancer	1.ORR 2.AE 3.SAE 4.PFS
4189		NCT05822752	Phase 2	Study to Evaluate Adverse Events, and Change in Disease Activity, When Intravenously (IV) Infused With Livmoniplimab in Combination With IV Infused Budigalimab in Adult Participants With Hepatocellular Carcinoma (HCC) (LIVIGNO-1)	120	Hepatocellular Carcinoma	1.BOR
4190		NCT06109272	Phase 2, Phase 3	A Study to Assess the Dose, Adverse Events, and Change in Disease Activity of Livmoniplimab as an Intravenous (IV) Solution in Combination With Budigalimab as an IV Solution in Adult Participants With Hepatocellular Carcinoma (HCC) (LIVIGNO-2)	660	Hepatocellular Carcinoma	1.BOR 2.OS
4191		NCT05733598	Phase 2	RP3 in Combination With 1L or 2L Therapy in Patients With Locally Advanced Unresectable or Metastatic HCC	60	Locally Advanced Hepatocellular Carcinoma, Recurrent Hepatocellular Carcinoma, Metastatic Hepatocellular Carcinoma	1.ORR
4192	BC2059	NCT05797805	Phase 1, Phase2	A Study of Tegavivint (BC2059) in Patients With Advanced Hepatocellular Carcinoma	108	Advanced Hepatocellular Carcinoma	1.TRAE 2.DLT 3.Evaluate efficacy
4193		NCT06172205	Phase 3	Infusional FOLFOX Plus Camrelizumab and Apatinib vs HAIC-FOLFOX Plus Camrelizumab and Apatinib for Advanced HCC	192	BCLC Stage C Hepatocellular Carcinoma, Chemotherapy Effect	1.ORR
4194		NCT05198609	Phase 3	Camrelizumab, Apatinib Plus HAIC Versus Camrelizumab and Apatinib for HCC With Portal Vein Invasion: a Randomized Trial	214	Hepatocellular Carcinoma	1.OS
4200	REPLACE	NCT04777851	Phase 3	Regorafenib-pembrolizumab vs. TACE/​TARE in Intermediate Stage HCC Beyond Up-to-7 (REPLACE)	496	Carcinoma, Hepatocellular	1.PFS

## ICIs

mDurvalumab, an anti-PD-L1 antibody, has been explored in various combinations for the treatment of unresectable HCC. A phase 2 trial (NCT02519348) evaluated durvalumab monotherapy and combinations with tremelimumab and bevacizumab (13). The study found that both the STRIDE regimen (Single Tremelimumab Regular Interval Durvalumab) and durvalumab plus bevacizumab (D+B) demonstrated higher objective response rates (ORR) compared to durvalumab alone. Notably, the STRIDE regimen exhibited significant immune modulation effects, indicating potential complementary actions when combined with durvalumab and bevacizumab. Another multi-institutional analysis focused on atezolizumab and bevacizumab (A+B) as a first-line systemic therapy for advanced HCC (14). Results showed that median overall survival (OS) was significantly better in patients with Child-Pugh A liver function compared to those with poorer liver function. Additionally, the albumin-bilirubin (ALBI) grade was found to be an effective predictor of patient outcomes, underscoring the importance of liver function scores in treatment planning. A real-world study from the HCC CHORD database compared atezolizumab plus bevacizumab (AB) versus lenvatinib (LEN) as first-line systemic therapy for HCC. AB was associated with superior OS compared to LEN (19.7 vs. 14.4 months) but had similar PFS and RR (15).

In the additional follow-up of the KEYNOTE-224 study investigating the efficacy of pembrolizumab in patients with advanced HCC previously treated with sorafenib, pembrolizumab exhibited durable responses with ORRs of 18.3% and 17.6% in sorafenib-treated and treatment-naive cohorts, respectively, with a manageable safety profile (16). A multicenter study found adjuvant immunotherapy improved recurrence-free and overall survival in intermediate/advanced HCC patients post-hepatic resection, compared to non-immunotherapy groups, highlighting benefits for high-risk patients (17). Moreover, in the NeoLEAP-HCC trial, a single-arm, multi-center, phase II study, pembrolizumab combined with lenvatinib was evaluated as a perioperative treatment for resectable HCC (18). The combination demonstrated promising anti-tumor efficacy, with 37.8% of patients achieving a major pathological response (MPR) and an acceptable safety profile, showing potential in reducing recurrence rates post-surgery. Tislelizumab, another PD-1 inhibitor, was studied in combination with intensity-modulated radiotherapy (IMRT) in a single-arm phase II trial for patients with resectable HCC and macrovascular invasion (MVI) (19). This study reported an ORR of 30.0% and a significant pathological response in 66.7% of patients who underwent surgery, indicating the combination could be effective and tolerable as perioperative therapy.

Meanwhile, a phase II trial investigated livmoniplimab (anti-GARP-TGF-b1) combined with budigalimab (anti-PD-1) in advanced HCC patients who progressed on first-line therapy. Early results showed a 42% ORR, indicating promising clinical activity and manageable safety (20). Additionally, a phase 1/2 study evaluated tegavivint, a TBL1 inhibitor, in advanced HCC patients with beta-catenin activating mutations, aiming to characterize the safety, pharmacokinetics/pharmacodynamics (PK/PD), and preliminary antitumor activity of tegavivint, offering a novel targeted approach for HCC with specific genetic profiles (21). The GEMINI-Hepatobiliary phase II trial evaluated novel immuno-oncology regimens for advanced hepatobiliary cancers, including HCC (22). This study explored the efficacy of volrustomig (anti-PD-1/CTLA-4) or rilvegostomig (anti-PD-1/TIGIT) in combination with standard anticancer agents. The trial aims to provide new insights into improving outcomes for advanced hepatobiliary cancers through innovative IO-based treatments.

A phase II/III study is investigating the combination of livmoniplimab and budigalimab in patients with locally advanced or metastatic HCC. The study aims to determine the optimal dose and evaluate the efficacy and safety of this novel combination, potentially offering a new therapeutic option for advanced HCC (23). Another randomized, open-label, phase III trial is comparing apatinib and camrelizumab with or without HAIC for HCC with portal vein tumor thrombus (PVTT). This study aims to determine the efficacy and safety of adding to the combination of apatinib and camrelizumab, potentially offering an enhanced treatment strategy for patients with high-risk HCC (24). A phase III trial (NCT05198609) is evaluating the efficacy and safety of apatinib and camrelizumab plus intravenous FOLFOX or HAIC with FOLFOX for advanced HCC. This trial seeks to compare the efficacy of these regimens, aiming to optimize treatment for advanced HCC (25).

A phase III study of SCT-I10A, an anti-PD-1 antibody, combined with a bevacizumab biosimilar versus sorafenib in advanced HCC demonstrated that SCT-I10A plus SCT510 showed significantly longer median OS (22.1 vs. 14.2 months) and PFS (7.1 vs. 2.9 months) compared to sorafenib (26). The combination also had a higher ORR with an acceptable safety profile. A phase II trial (NCT04039607) is assessing nivolumab and ipilimumab versus Lenvatinib or sorafenib as first-line treatment for unresectable HCC. Nivolumab plus ipilimumab showed a significant OS benefit, higher ORR, and durable responses, suggesting it as a potential new first-line standard of care for unresectable HCC (27). Lastly, the final OS analysis of the study CARES-310 showed that Camrelizumab plus rivoceranib had a median OS of 23.8 months compared to 15.2 months for sorafenib, demonstrating superior efficacy (28). An international phase II study evaluated regorafenib and pembrolizumab in advanced HCC previously treated with ICIs (29). The study reported modest activity, with an ORR of 5.9% and a median PFS of 2.8 months, suggesting benefits for selected patients who progressed on prior ICI treatment.

ICIs have shown improvements in OS and PFS, particularly when combined with agents like bevacizumab. However, patient response remains variable, with factors such as liver function and biomarkers like PD-L1 expression influencing outcomes. irAEs are a notable concern, often requiring intensive management. Additionally, resistance mechanisms may limit long-term efficacy. Future strategies should focus on identifying reliable biomarkers to guide patient selection and combining ICIs with other therapies to mitigate resistance and improve outcomes.

## Locoregional treatment-related therapies

Locoregional treatments including TACE and HAIC have been playing an important role in HCC treatment. Additionally, using locoregional treatment to activate antitumor immunity has been a useful way to enhance the efficacy of ICIs. The phase III EMERALD-1 study investigated durvalumab with or without bevacizumab combined with TACE in unresectable HCC. The combination significantly improved PFS compared to placebo plus TACE, supporting its potential as a new standard of care for embolization-eligible HCC​ (30). A phase II study investigated the combination of envafolimab, an anti-PD-L1 antibody, with lenvatinib and TACE in initially unresectable HCC (31). The combination demonstrated promising survival outcomes, with a median PFS of 8.78 months and a manageable safety profile, indicating its potential as a conversion therapy for surgical resection. A single-arm, phase II trial (PLATIC) evaluated sintilimab, lenvatinib, and TACE-HAIC as conversion therapy for initially unresectable HCC. The study reported a 77.2% conversion to resection rate, with an ORR of 77.2% (mRECIST) and 42.1% (RECIST 1.1). The mPFS was 14.3 months. Grade 3/4 TRAEs occurred in 64.9% of patients (32).

A phase II trial assessed HAIC with tislelizumab and lenvatinib in unresectable HCC. The study showed efficacy, with a median PFS of 15 months and a high conversion to resection rate, highlighting the potential for downstaging advanced HCC (33). A phase III trial compared sorafenib plus HAIC with sorafenib plus TACE in advanced HCC (34). Results showed HAIC with sorafenib significantly improved OS and PFS compared to TACE with sorafenib, especially benefiting patients with high fatty acid degradation (FAD) activity. Additionally, a phase III trial (REPLACE) is comparing the efficacy and safety of regorafenib and pembrolizumab versus locoregional therapy (TACE/TARE) for intermediate-stage HCC beyond the up-to-7 criteria, addressing the need for effective systemic therapy in intermediate-stage HCC (35). A phase II trial evaluated venous infusion chemotherapy (VIC) combined with apatinib and camrelizumab for CNLC stage III HCC. The study reported a confirmed ORR of 60.0%, a disease control rate of 97.1%, and a manageable safety profile, indicating significant anti-tumor effects (36). Another study compared stereotactic body radiotherapy (SBRT) combined with TACE and tyrosine kinase inhibitors (TKIs) versus TACE and TKIs alone in HCC patients with PVTT. Results showed that the SBRT+TACE+TKIs group had significantly better progression-free survival (PFS) and overall survival (OS), suggesting improved efficacy without additional safety concerns (37).

Locoregional treatments like TACE and HAIC, when combined with ICIs or TKIs, have improved tumor control and, in some cases, allowed resection of previously unresectable tumors. However, their efficacy is closely tied to liver function, and recurrence rates remain high. Procedural variability and differences in embolization techniques contribute to inconsistent outcomes. Standardization of treatment protocols and optimization of combinations with systemic therapies will be crucial in improving long-term results.

## CAR-T cell therapies

C-CAR031, a GPC3-specific TGFbRIIDN armored autologous CAR-T therapy, was evaluated in a phase I study for advanced HCC (38). The treatment exhibited a manageable safety profile, with an ORR of 50% and a median PFS of 4.27 months. These preliminary results indicate promising efficacy for CAR-T cell therapy in heavily treated advanced HCC patients, offering a new potential treatment modality. However, challenges persist in solid tumor settings. Tumor microenvironment factors, such as immunosuppression, limit CAR-T cell persistence and efficacy. Furthermore, managing toxicities like CRS and neurotoxicity is critical. Although the high cost and complexity of CAR-T production are obstacles, further refinement in CAR-T engineering could enhance their role in treating advanced HCC.

## Novel oncolytic virus therapies

VG161, an oncolytic virus expressing IL12, IL15, and PD-L1 blocking peptide, was evaluated in a phase I trial for HCC patients refractory to two prior lines of therapy (39). The study demonstrated a 17.14% ORR and a 60.00% disease control rate (DCR), with a median OS of 9.40 months. These results suggest that VG161 has the potential to benefit heavily pre-treated HCC patients, offering a new therapeutic approach. A multicenter phase II trial assessed the combination of oncolytic immunotherapy RP2 with atezolizumab and bevacizumab in advanced HCC. The study aims to evaluate the safety and efficacy of this combination as a second-line treatment, with primary endpoints including ORR and progression-free survival​ (40). Oncolytic viruses offer a novel approach with potential in patients who have failed other treatments. However, response rates have been modest, and improvements in delivery mechanisms and immune activation are needed. Combining oncolytic viruses with ICIs or other systemic therapies may boost their efficacy, but further large-scale trials are necessary to validate these combinations.

## Exploration of biomarkers for targeted therapies

Biomarkers hold significant potential for personalizing cancer treatment. However, significant challenges remain in integrating biomarker-driven approaches into routine clinical practice, particularly in HCC, where heterogeneity in both tumor biology and liver function complicates treatment decisions. Examples from other cancers provide valuable insights into the potential application of biomarker-driven therapies in HCC. For instance, in non-small cell lung cancer (NSCLC), the presence of EGFR mutations or ALK rearrangements has successfully guided the use of targeted therapies, dramatically improving outcomes (41, 42). Similarly, in melanoma, the identification of BRAF V600E mutations has led to the development of targeted therapies like vemurafenib, which have significantly extended survival in patients with this specific mutation (43, 44). These examples demonstrate the profound impact of biomarker-driven treatment on improving patient outcomes when the right molecular target is identified.

An analysis of RNA expression-based hypoxia scores (HS) identified it as a significant prognostic biomarker in HCC (45). The study revealed that tumors with high hypoxia scores (HS-high) had worse OS but were more likely to respond to immunotherapy. In contrast, tumors with low hypoxia scores (HS-low) showed better OS and responded more favorably to sorafenib. These findings suggest that the hypoxia score could guide personalized treatment strategies for HCC, enabling more tailored and effective approaches.

A multicenter analysis from the HCC-CHORD consortium examined predictors of short-term death (STD) and long-term survival (LTS) in advanced HCC patients treated with TKIs or immunotherapy (46). The study identified several key predictors: higher ALBI grade, microvascular invasion (MVI), and distant metastases were linked to increased short-term death risk, while lower ALBI grade and absence of MVI were associated with better long-term survival.

An exploratory analysis from the KEYNOTE-240 trial found that CTNNB1 mutations did not significantly impact pembrolizumab outcomes in advanced HCC (47). However, another investigation into CTNNB1 mutations in HCC patients revealed that lower CTNNB1 expression was associated with improved overall survival (OS), particularly in patients treated with ICIs or tyrosine kinase inhibitors (TKI), highlighting the potential of CTNNB1 expression as a biomarker for treatment selection (48). A *post-hoc* analysis from the REFLECT trial investigated ctDNA mutations in HCC patients treated with lenvatinib or sorafenib. Common mutations included TERT, TP53, and CTNNB1, with TP53 mutations linked to overall survival. This highlights the need for further research on these mutations’ impact on treatment outcomes (49). Another study showed that tumor-informed ctDNA is a reliable biomarker for detecting minimal residual disease (MRD) and guiding adjuvant therapy in HCC, enhancing postoperative management precision (50).

Targeted therapies, particularly TKIs, have shown efficacy but are limited by the development of resistance, often due to tumor heterogeneity. The use of biomarkers, such as hypoxia scores and ctDNA, may help personalize therapy and optimize outcomes. Combination strategies with locoregional treatments or immunotherapies may improve efficacy, but further research is needed to refine these approaches and address resistance mechanisms.

## Conclusion

The therapeutic landscape of HCC continues to evolve, with significant advancements across various treatment modalities. ICIs have demonstrated notable improvements in survival outcomes, especially in combination therapies, though variability in patient responses and immune-related adverse events (irAEs) remain key challenges. Locoregional treatments like TACE and HAIC play a crucial role, particularly in combination with systemic therapies. CAR-T therapies, while promising, face significant hurdles in the solid tumor microenvironment and require further refinement in delivery and toxicity management. Oncolytic viruses offer potential as novel therapies, though their response rates remain modest, and targeted therapies, particularly TKIs, are hindered by the development of resistance. Future efforts should focus on optimizing combination therapies, improving patient selection with biomarker-driven approaches, and addressing the challenges of treatment resistance and adverse effects to maximize the benefits of these innovations for HCC patients.
